# Modeling Innate Immunity Causing Chronic Inflammation and Tissue Damage

**DOI:** 10.1007/s11538-024-01410-0

**Published:** 2025-01-23

**Authors:** Kosei Matsuo, Yoh Iwasa

**Affiliations:** Department of Biology, Faculty of Science, Kyushu University, 744 Motooka, Nishi-Ku, Fukuoka, 819-0395 Japan

**Keywords:** Chronic inflammation, Pathogen infection, Tissue damage, Perpetual oscillation, Non-inflammatory immune activation

## Abstract

Mathematical models of immune responses have traditionally focused on adaptive immunity and pathogen-immune dynamics. However, recent advances in immunology have highlighted the critical role of innate immunity. In response to physical damage or pathogen attacks, innate immune cells circulating throughout the body rapidly migrate from blood vessels and accumulate at the site of injury, triggering inflammation. These cells engulf, break down, and eliminate pathogens. This innate immune response occurs much faster than adaptive immune responses, which require time for cell activation and proliferation. While inflammation helps eliminate pathogens, it can sometimes lead to chronic inflammation by triggering excessive immune responses, ultimately causing tissue damage. In this study, we examine a simple dynamical model of innate immunity. The analysis indicates that when an infection occurs, it triggers inflammation, which activates the innate immune system and initiates the activation cycle. Consequently, pathogens may be eradicated, leaving behind persistent chronic inflammation. Alternatively, the pathogens may not be eradicated, with their abundance either stabilizing at a positive level or oscillating indefinitely. The dynamics exhibit both transcritical and Hopf bifurcations. When innate immunity is activated in the absence of inflammation, pathogens are eradicated more easily, and the likelihood of oscillations in inflammation, immune responses, and pathogen abundance is reduced.

## Introduction

Mathematical models of immune responses have traditionally focused on adaptive immunity and pathogen-immune dynamics. In response to pathogen invasion, antigen-specific immune cells—such as helper T cells, cytotoxic T lymphocytes, and B cells—are activated and proliferate, ultimately suppressing pathogen growth. Dynamical models of the immune system's interaction with pathogens and analogous to predator–prey systems in ecology, while models incorporating the emergence of mutant strains that evade immune responses are somewhat similar to those in population genetics (e.g., Nowak and May 2000; Grenfell et al. 2004).

However, recent advances in immunology have revealed that the innate immune system plays a critical role (Parham 2021). The process operates as follows: When pathogens or cellular damage occur at a peripheral site in the body, innate immune sensor cells, such as macrophages and dendritic cells, first detect the abnormality. These cells possess pattern recognition receptors (PRRs) that identify pathogen-associated molecular patterns (PAMPs) or damage-associated molecular patterns (DAMPs). In response, the immune sensor cells release inflammatory mediators, such as cytokines and chemokines, which cause blood vessels to dilate, increase blood flow, and raise vessel permeability. The released chemokines signal circulating neutrophils, macrophages, basophils, other myeloid cells, and natural killer cells to migrate toward the source of the signals and accumulate at the site of the problem. There, they engulf, break down, and eliminate the pathogens. Once the issue is resolved, the inflammatory response is suppressed, and the immune cells gradually leave the site, bringing the inflammation to an end. This innate immune response is very rapid and serves as the body's first line of defense against pathogen invasion or physical damage.

While inflammation helps eliminate pathogens, excessive inflammation can be harmful, potentially leading to further tissue damage and adverse effects. To prevent excessive tissue damage from overly strong inflammation, a self-regulating mechanism known as the anti-inflammatory response occurs (Yasukawa et al. 2003; Lacatus 2013). Chronic inflammation refers to a condition in which the inflammatory response persists for a prolonged period within the body. It often occurs as individuals age and can lead to various diseases (Shaw et al. 2013; Franceschi and Campisi 2014; Calçada et al. 2014; Ray and Yung 2018; Singh et al. 2019; Öberg et al. 2021).

Chronic inflammation typically arises when acute inflammation is not resolved or when persistent stimuli exist. It can have significant implications for health, often signaling an imbalance in the body's immune system. This imbalance may lead to the development of diseases such as autoimmune conditions or allergic reactions (McInnes and Schett 2007). Additionally, chronic inflammation is believed to play a role in the development and progression of chronic diseases such as cancer, cardiovascular diseases, and diabetes (Hasselbalch 2012). Furthermore, it can result in tissue damage and functional impairment. For example, in rheumatoid arthritis, persistent inflammation of the joints can result in joint deformities and functional disabilities. Finally, chronic inflammation can manifest in systemic symptoms and discomfort, including fatigue, lethargy, joint pain and swelling, and fever.

Inflammation may initiate after infection with a pathogen and persist even after the pathogen has been eliminated, particularly when the immune system overreacts and inflammation is not properly controlled. This phenomena is known as "excessive inflammation response," which can potentially lead to tissue or cell damage due to sustained inflammation (Chu et al. 2017). In autoimmune diseases such as rheumatoid arthritis or ulcerative colitis, the immune system mistakenly identifies the body's own tissues as foreign and triggers an excessive inflammatory response (Baker et al. 2013; Ordás et al. 2012). As a result, inflammation persists even after the pathogen has been cleared, potentially causing damage to tissues and organs. Additionally, chronic infections or allergic reactions may also lead to continued inflammation even after the pathogen has been eliminated. In such cases, the immune system displays an excessive response, resulting in persistent inflammation.

In this study, we examine a simple mathematical model that depicts the interaction between innate immunity, chronic inflammation, and tissue damage. We focus on the dynamical interaction between innate immunity and inflammation, addressing phenomena before the onset of adaptive immunity. If the pathogens are not eradicated by innate immunity and remain in the body, adaptive immunity begins to function, and the dynamics must incorporate immune cells responsible for antigen-specific responses (e.g., helper T cells, cytotoxic T cells, B cells), which is beyond the scope of the current paper. The analysis of the dynamics reveals that, depending on parameters, the model may demonstrate activation cycles of innate immunity, inflammation, and tissue damage, potentially resulting in the eradication of the pathogen while leaving behind persistent chronic inflammation. The model may also exhibit the persistence of pathogens at a constant level or oscillation. The degree of tissue damage reaches its minimum when the levels of inflammation and non-inflammatory immune activation are intermediate.

## Model

**Fig. 1:**
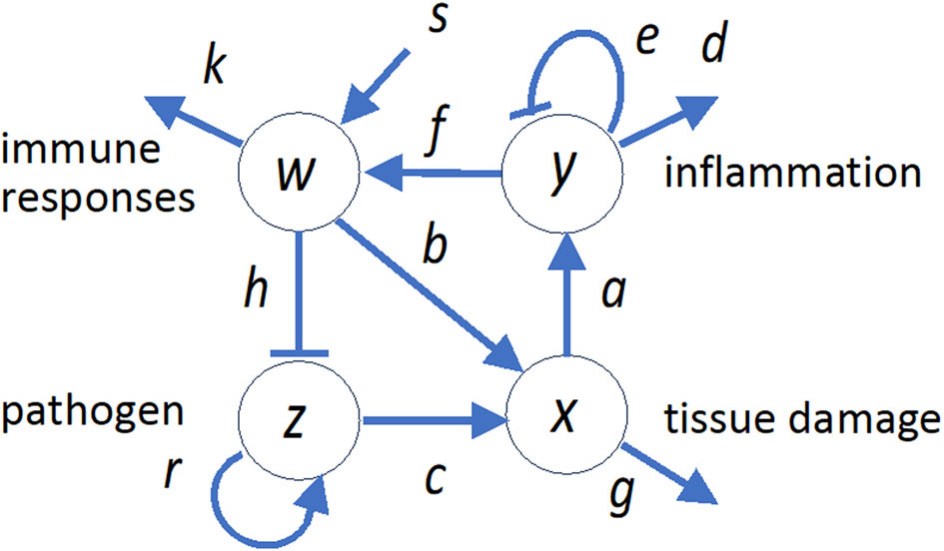
Scheme of the model. Four variables are depicted: pathogen abundance $z$; immune responses $w$; inflammation $y$; and tissue damage $x$. Arrows indicate interactions between them. Refer to the text for further explanation

Here, we examine a system of differential equations involving four variables: the strength of the immune response $w$, the severity of tissue damage $x$, the amount of inflammatory cytokines $y$, and the abundance of pathogens $z$. Figure 1 illustrates the interaction between the variables. We represent their dynamics and interaction by the following differential equations.

Let us consider a portion of peripheral region. We represent the strength of innate immunity as the number of innate immune cells (e.g., neutrophils, basophils) at the focal site. These cells are circulating the body, and when inflammation cytokines are released, they come out of blood vessels and accumulate at the focal site. On the other hand, the innate immune responses diminish in the absence of new immune stimulation by inflammatory cytokines, decaying exponentially. The dynamics of immune response are described by the following equations:

1a $$\frac{dw}{dt}=fy-kw$$

For simplicity, we first consider the case where the activation rate of innate immune strength is proportional to the amount of inflammatory cytokines $y$. The first term on the right-hand side of Eq. (1a) represents the enhancement of the immune response by inflammatory cytokines, with a proportionality coefficient $f$. The second term indicates the decay of immune strength, where $k$ represents the rate of disappearance of immune cells due to mortality or dispersal. We will examine a different functional form for the activation rate later, where we consider both inflammatory and non-inflammatory rates of immune activation.

The presence of pathogens damages tissues, but the immune system can also cause injury to tissues during its operation. We assume that the observed tissue damage, denoted as $x$, increases with the sum of a term proportional to the strength of immune function, $w$, and a term proportional to the quantity of pathogens, $z$. The dynamics of the tissue damage level are given as follows:

1b $$\frac{dx}{dt}=bw+cz-gx$$

In this equation, the first term on the right-hand side signifies tissue damage associated with immune attacks, the second term represents tissue damage caused by pathogens, and the third term stands for the rate of recovery from damages. The coefficients $b$, $c$, and $g$ represent the rates of these processes.

We assume that the dynamics of the inflammatory cytokines, denoted as $y$, are described by the following equation:

1c $$\frac{dy}{dt}=ax-\left(d+ey\right)y$$

The first term on the right-hand side indicates that inflammatory cytokines production is proportional to the damage inflicted on tissues. The second term represents the effect of inflammatory cytokines naturally subsiding, with suppression becoming stronger as inflammation $y$ increases in an accelerating manner, representing anti-inflammatory responses. This may be realized by inhibitory cytokinin or any transcriptional regulation. As a consequence, while the level of inflammatory cytokines produced $y$ increases with increasing damage $x$, it does not increase proportionally; instead, the rate of increase in $y$ diminishes as $y$ increase (tapering off). It's worth noting that this behavior could also be expressed by assuming nonlinearity in the first term. However, here, it's represented by expressing the second term as a function that increases faster than proportionally. In Eq. (1c), $a$ denotes induction of inflammatory cytokines based on signals from damage, $d$ represents the degradation of inflammatory cytokines, and $e$ signifies self-inhibition through self-feedback of inflammation.

Finally, the abundance of pathogens $z$ in the focal tissue changes over time according to the following equation:

1d $$\frac{dz}{dt}=rz-hwz$$

If the immune system is not functioning ($w=0$), pathogens increase exponentially within the host organism, where $r$ denotes the exponential rate of pathogen proliferation. The second term on the right-hand side represents the suppression exerted by the immune system's activity, where $h$ represents immune attacks on pathogens. This functional form has been adopted in many models of host–pathogen dynamics, such as those concerning viral dynamics within a host (Nowak and May 2000).

### Examples of the Dynamics

**Fig. 2:**
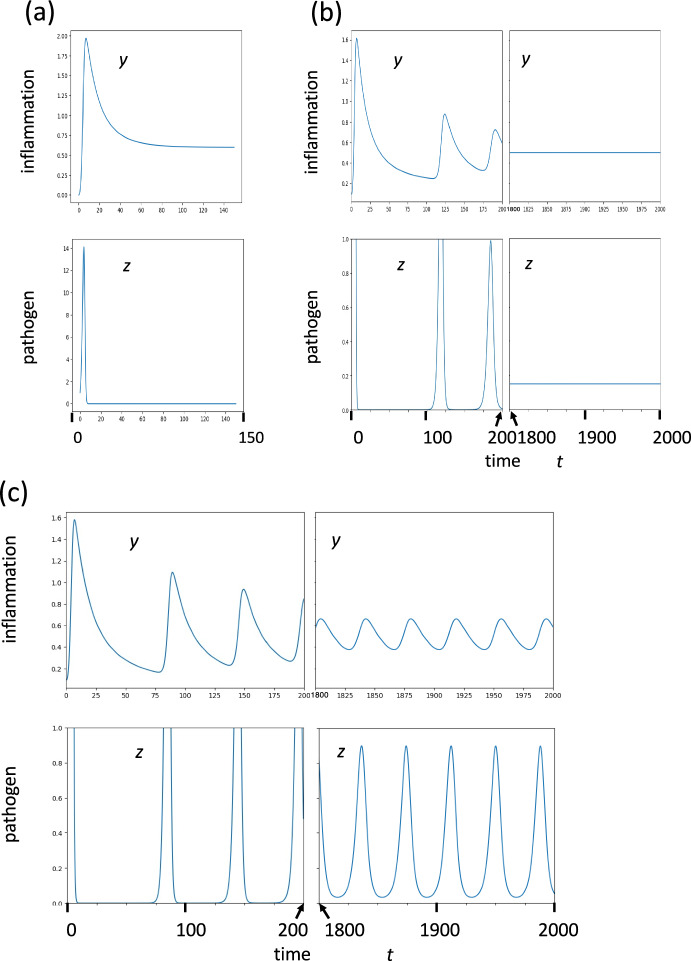
Dynamics of the model in three typical cases. (**a**) $d=0.08$. Pathogens are eventually eradicated (Case I). (**b**) $d=0.16$. Pathogen abundance converge to a positive level (Case II). (**c**) $d=0.205$. Pathogen abundance keeps fluctuating indefinitely (Case III). Other parameters are: $a=0.2$, $b=0.2$, $c=0.2$, $e=0.2$, $f=2$, $g=0.2$, $h=2$, $k=2$, $r=1$, and $s=0$

Figure 2 illustrates several examples of the dynamics of the model. In Fig. 2a, when the pathogen invaded with a small initial dose, its abundance temporarily increased to a high level. However, due to the function of immune reactions, the pathogen abundance z decreased and finally it converges to zero indicating its eradication (see the upper panel of Fig. 2a). The lower panel of Fig. 2a illustrates the dynamics of inflammation, $y$. Before the infection, $y$ was 0. When the host was infected by the pathogen, inflammation $y$ increased together with tissue damage $x$ and innate immunity $w$. However, the inflammation remained at a high level even after the pathogen was eradicated from the body, implying chronic inflammation.

In the case illustrated in Fig. 2b, the pathogen did not disappear, but its abundance converged to a positive level. Hence, pathogen remained in the body forever. The inflammation $y$, immune response $w$, and tissue damage $x$ all remained positive.

In the case illustrated in Fig. 2c, the pathogen remained in the body but did not converge to a stationary level. Unlike the scenario in Fig. 2b, where the pathogen abundance $z$ stabilized, in Fig. 2c, $z$ continued to oscillate perpetually. Similarly, the levels of inflammation $y$, immune reaction $w$, and tissue damage $x$ also exhibited continuous fluctuations.

In the sections below, we investigate the conditions that give rise to different behaviors in the dynamics.

## Equilibria

The equilibria are obtained by setting the right-hand sides of Eqs. (1a), (1b), (1c) and (1d) equal to zero. From Eq. (1d), we identify two types of equilibria:

- [Type 1] $z=0$. Pathogen is eradicated from the body.
- [Type 2] $z>0$ and $w=\frac{r}{h}$. Some pathogens are maintained in the body.

In Appendix A, we investigate the equilibria and their stability. The results are summarized as follows:

There are two parameter regions which affect the behavior of the dynamics differently.

[Region I] When $d+e\frac{rk}{hf}<\frac{abf}{gk}$ holds, there exists a single equilibrium without pathogen ($z=0$) that is globally stable. This indicates that the system will converge to the equilibrium starting from any initial state. The state variables at the equilibrium are given as follows:

2 $$ z = 0,\;y = \frac{1}{e}\left( {\frac{abf}{{gk}} - d} \right),\;x = \frac{bf}{{gke}}\left( {\frac{abf}{{gk}} - d} \right)\;{\text{and}}\;w = \frac{f}{ke}\left( {\frac{abf}{{gk}} - d} \right) $$

The levels of inflammation, tissue damage, and immune activity decrease with $d$ and are proportional to each other. At the equilibrium, the immune activity enhanced by inflammation makes the eradication of pathogen possible, as illustrated in Fig. 2a. There is no equilibrium with a positive abundance of the pathogen in parameter region I.

[Region II] When $d+\frac{erk}{hf}>\frac{abf}{gk}$ holds, a single equilibrium with nonzero abundance of the pathogen exists.

At equilibrium, the state variables are given as follows:

3 $$ z = \frac{1}{c}\left( {\frac{g}{a}\left( {d + \frac{erk}{{hf}}} \right)\frac{rk}{{hf}} - \frac{br}{h}} \right),\;y = \frac{rk}{{fh}},\;x = \frac{1}{a}\left( {d + \frac{erk}{{fh}}} \right)\frac{rk}{{fh}},\;{\text{and}}\;w = \frac{r}{h} $$

$z>0$ holds in parameter region II.

In this region, there also exists an equilibrium without pathogen ($z=0$), which is unstable, as discussed in the next section. The equilibrium with some pathogen, if it exits, may be either stable or unstable, which is also discussed in the next section.

**Fig. 3:**
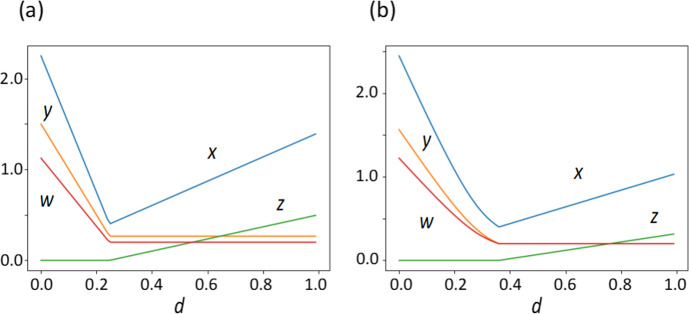
Values of four state variables at the equilibrium. Tissue damage (blue); inflammation (orange); immune responses (red); pathogen abundance (green). The horizontal axis indicates parameter $d$. (**a**) Non-inflammatory activation is absent ($s=0$). (**b**) Some non-inflammatory activation exists ($s=0.1$). Other parameters are: $a=0.2$, $b=0.2$, $c=0.2$, $e=0.2$, $f=1.5$, $g=0.1$, $h=2$, $k=2$, and $r=0.4$

Figure 3a illustrates the value of four variables at the equilibrium changing with parameter $d$. Let ${d}_{1}=\frac{abf}{gk}-\frac{erk}{hf}$. For $d<{d}_{1}$, the only equilibrium is given by Eq. (2). No pathogen exists ($z=0$), and all three variables, $w, x$, and $y$ increase with $d$, as indicated by Eq. (2). For $d>{d}_{1}$, there are two equilibria: one given by Eq. (3) that is stable and the other given by Eq. (2) that is unstable. (The stability of the.

equilibrium is discussed in the subsequent section and in Appendix B.) At the stable equilibrium, some pathogens remain ($z>0$), and $w$ and $y$ are constant but tissue damage $x$ and pathogen $z$ increases with $d$. Since $d$ is the decay rate of inflammation, larger $d$ implies weaker inflammation. As $d$ increases from 0, the strength of inflammation decreases, leading to a decrease in excess immune activity and the tissue damage caused by immune activity. At $d={d}_{1}$, no pathogen exists, but inflammation $y$ immune activity $w$ and harm $x$ are at positive levels. As $d$ further increases, the pathogen abundance increases, and the harm becomes stronger. Note that tissue damage reaches its minimum at the boundary between these two regions ($d={d}_{1}$).

### Stability of the Equilibria

**Fig. 4:**
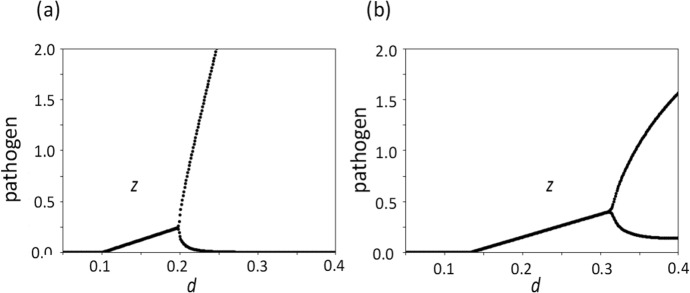
Bifurcation diagram. The horizontal axis indicates $d$, and the vertical axis indicates pathogen abundance in the final value $z$. (**a**) For $d<{d}_{1}$, the equilibrium with some pathogen does not exist. The equilibrium with $z=0$ is globally stable. For ${d}_{1}<d<{d}_{2}$, the equilibrium with some pathogen exists, and it is stable. All trajectories converge to the stationary state with a positive abundance of the pathogen. There is the equilibrium with $z=0$, which is unstable. For $d>{d}_{2}$, the equilibrium with some pathogen exists, but it is locally unstable and is surrounded by a stable limit cycle. The system oscillates perpetually, and as $d$ increases, the amplitude of oscillation becomes larger. In the present figure, we have ${d}_{1}=0.1$ and ${d}_{2}=0.2$. At $d={d}_{1}$ and $d={d}_{2}$, a transcriptional bifurcation and a supercritical Hopf bifurcation take place, respectively. (**b**) This figure is the same as in (**a**), except that non-inflammatory activation of immunity exists ($s=0.1$). Both ${d}_{1}\approx 0.13$ and ${d}_{2}\approx 0.32$ are larger than the corresponding values in (**a**). Other parameters are: $a=0.2, b=0.2, c=0.2, e=0.2, f=2, g=0.2, h=2, k=2,$ and $r=1$

Figure 4a illustrates the bifurcation diagram. The horizontal axis represents $d$, the rate of suppression of inflammation. The vertical axis indicates the pathogen abundance $z$ that is achieved after a sufficiently long time from the initial state. For $d<0.1$, the pathogen is eradicated from the body $z=0$. For $0.1<d<0.2$, pathogen abundance converges to a positive level $z>0$, indicated by the thick curve.

For $d>0.2$, pathogen abundance oscillated perpetually. Here, we illustrate the oscillation by showing the maximum and the minimum values of $z$. Two critical values of $d$ are denoted as ${d}_{1}=0.1$ and ${d}_{2}=0.2$ in Fig. 4a.

The numerical results suggest that, as the parameter $d$ changes continuously around $d={d}_{1}$, the equilibrium $z=0$ transitions from being globally stable to unstable. Another equilibrium with a positive $z$ splits and becomes globally stable. This indicates a transcritical bifurcation (or alternation of stability) at $d={d}_{1}$ (Strogatz 1994).

In contrast, as $d$ increases from below $d={d}_{2}$ to above it, the globally stable equilibrium becomes an unstable equilibrium, which is surrounded by a stable limit cycle. This indicates a super-critical Hopf bifurcation (Strogatz 1994).

#### Phase Plane

**Fig. 5:**
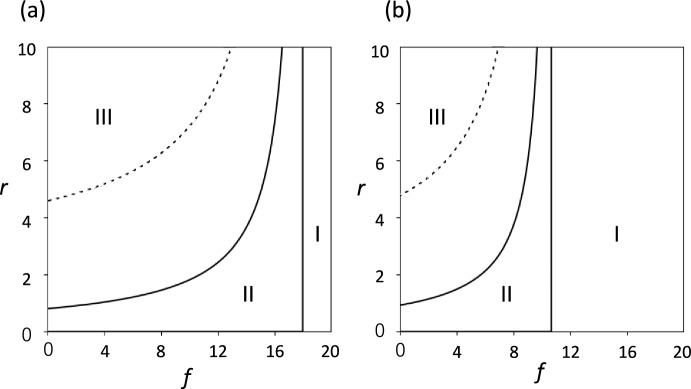
Phase plane showing three different behaviors, as illustrated in Fig. 2. Parameter regions for three different outcomes are indicated: (I) pathogen is eradicated; (II) pathogen persists at a positive stationary level; (III) pathogen persist with the abundance oscillating perpetually. We changed $f$ and $e$ with their ratio fixed: $e=0.5f$. We also changed $r$ and $h$ with their ratio fixed: $h=r$. The horizontal and the vertical axes indicate $f$ and $r$, respectively. Each curve indicates the boundary between parameter regions for perpetual oscillation (III) and stationary state (II). A solid curve represents the original four-variable dynamics, while a broken curve is for the reduced three-variable dynamics. (**a**) Non-inflammatory activation is absent ($s=0$). (**b**) Some non-inflammatory activation is present ($s=0.5$). Other parameters are: $a=0.2$, $b=0.2$, $c=0.2$, $d=0.2$, $g=0.3$, and $k=2$

Figure 5a illustrates the phase plane for three qualitatively different outcomes: Region I represents the parameter space where pathogens are eradicated; Region II indicates where pathogens are maintained at a positive stationary level; Region III indicates perpetual oscillation of pathogen abundance. The horizontal axis represents $f$ with $e$ being proportional to $f$ (i.e., $e=0.5f$). The vertical axis represents $r$, with $h$ being proportional to $r$. All other parameters are held fixed (refer to the caption to Fig. 5).

#### Condition for Oscillation in Reduced Three-Variable Model

The variable $w$ represents the strength of innate immune responses at the focal region. It may increase by inflammation because the immune cells, such as neutrophils or macrophages, are attracted from the rest of the body to the focal region. Since this requires less time than cell division, cell differentiation, or activation, the variable can change rather quickly. We can assume that the immune strength $w$ changes more quickly than the other three variables ($x, y, z$). Based on this approximation of differences in the speed of changes, we can simplify the dynamics.

We consider the case in which $f$ and $k$ are larger than the other rate parameters. Then, the fast variable $w$ converges to a quasi-equilibrium while the other three variables remain fixed. From Eq. (1a), we have $0=fy-kw$, which leads to $w=\frac{f}{k}y$. If $w$ is replaced by this formula, Eqs. (1b), (1b), and (1c) gives a reduced dynamics of three slow variables. The equilibria of the three-variable dynamics are the same as the four-variable model, Eqs. (1a), (1b), (1c), and (1d) and are given in Eqs. (2) and (3). However, the stability of the equilibria can be different.

In Appendix A, we demonstrate that the equilibrium with a pathogen ($z>0$) exists if and only if $z$ given by Eq. (3) is positive, which is equivalent to the inequality condition for the equilibrium without pathogen ($z=0$) being unstable, as shown in Appendix B. Hence, in the parameter range where the boundary equilibrium $z=0$ is stable, the equilibrium with a pathogen $z>0$ does not exist. Conversely, when the boundary equilibrium is unstable, the equilibrium with a pathogen exists. This implies that a transcritical bifurcation (or alternation of stability) takes place. For a more detailed discussion, please refer to Appendix B.

In Appendix B, we derive the condition for the local stability of the equilibrium with some pathogen as follows:

4 $$\left(g+d+2\frac{erk}{hf}\right)\left(\left(d+2\frac{erk}{hf}\right)g-\frac{abf}{k}\right)>\frac{hf}{k}ac\frac{1}{c}\left(\frac{g}{a}\left(d+\frac{erk}{hf}\right)\frac{rk}{hf}-\frac{br}{h}\right)=\left(gr\left(d+\frac{erk}{hf}\right)-\frac{abfr}{k}\right)$$

Numerical analyses confirm its global stability. Conversely, if the inequality opposite to the stability condition holds, the equilibrium is unstable. It becomes surrounded by a stable limit cycle, indicating perpetual oscillation, which acts as an attractor. The numerical analyses validate the stability condition predicted by the above inequality.

In Appendix B, we also discussed the condition for the local stability of the equilibrium for the four-variable model, given by Eqs. (1a), (1b), (1c), and (1d), but found it to be more complex than the three-variable model. Upon comparison, we observed that the condition for the transcritical bifurcation remains the same between the reduced dynamics with three variables and the original four-variable model.

However, the stability condition for the equilibrium with $z>0$ differs between the models. The parameter region for oscillation is broader in the four-variable models than in the reduced three-variable model. This difference is clearly illustrated in the phase plane shown in Fig. 5a. It suggests that the time-delay in the response of the immune system to be activated tends to make oscillation more likely.

## Innate Immunity with Non-inflammatory Activation

In this section, we consider the case in which the rate of activation of innate immunity remains positive even in the absence of pathogens or inflammation. An important characteristic of innate immunity is the presence of a baseline level of immune activation; even before pathogen infection and tissue damage. The strength of innate immunity at the focal tissue $w$ follows the dynamics:

5 $$\frac{dw}{dt}=s+fy-kw$$

instead of Eq. (1a). In the right-hand side of Eq. (5), $s$ represents the non-inflammatory rate of immune activation and reflects the basic defense provided by innate immunity. Without inflammation ($y=0$), innate immunity is maintained at a positive level, $w=\frac{s}{k}$. Inflammation in a portion of tissue attracts immune cells from other parts of the body and strengthens the immune activity at the focal point. $f$ represents the effectiveness of inflammation in enhancing immunity of the focal location.

The set of differential equations, including Eqs. (5), (1b), (1c), and (1d), forms the system we study in this section. By examining them and comparing their behavior with those in the model studied so far ($s=0$), we can tell the effect of the basic defense provided by innate immunity $s$.

### Equilibria and Their Stability

We obtain the four equations at the equilibrium by setting Eqs. (5), (1b), (1c) and (1d) to zero. From Eq. (1b), either $z=0$ or $hw=r$ holds. According to the analyses in Appendix A, we have the following results:

(i) Equilibrium without pathogen ($z=0$)
  As explained in Appendix A, we have the following values at equilibrium:
  6a $$z=0$$
  6b $$y=\frac{1}{2e}\left(\frac{abf}{gk}-d+\sqrt{{\left(\frac{abf}{gk}-d\right)}^{2}+4e\frac{abs}{gk}}\right)$$
  Two other variables are calculated from Eq. (6b) as follows:
  6c $$w=\frac{1}{k}\left(s+fy\right)$$
  6d $$x=\frac{b}{gk}\left(s+fy\right)$$
  These equations are slightly more complicated than Eq. (2), but they decrease with $d$ similarly to Eq. (2).
  If we increase $s$ with all the other parameters fixed, $y$, $w$, and $x$ increase with $s$ according to Eqs. (6b, 6c, 6d). Hence, in the absence of a pathogen, the equilibrium level of tissue damage $x$ and inflammation $y$, as well as immune responses $z$, increase with the non-inflammatory activation $s$.
  In Fig. 3b, the value of $y$ is not a straight line but slightly curved, because Eq. (6b) is not a linear function of $d$. Hence, in Fig. 3b for small $d$, neither $w$ nor $x$ are straight lines.
(ii) Equilibrium with pathogen ($z>0$)
  At equilibrium with $z>0$, we have the following solution:
  7a $$w=\frac{r}{h}$$
  7b $$y=\frac{1}{f}\left(k\frac{r}{h}-s\right)$$
  7c $$x=\frac{1}{a}\left(d+ey\right)y=\frac{1}{a}\left(d+e\frac{1}{f}\left(k\frac{r}{h}-s\right)\right)\frac{1}{f}\left(k\frac{r}{h}-s\right)$$
  7d $$z=\frac{1}{c}\left(gx- b\frac{r}{h}\right)=\frac{1}{c}\left(g\frac{1}{a}\left(d+e\frac{1}{f}\left(k\frac{r}{h}-s\right)\right)\frac{1}{f}\left(k\frac{r}{h}-s\right)- b\frac{r}{h}\right)$$

Variables $w$ and $y$ are independent of $d$, but $x$ and $z$ increase with $d$. This behavior is given by Eqs. (7a, 7b, 7c, 7d) for large $d$ (see Fig. 3b).

As $s$ increases from 0, all $x$, $y$, and $z$ decrease. Among them, pathogen abundance $z$ first reaches zero while both $x$ and $y$ are still positive. We denote the value of $s$ which realizes $z=0$ with Eq. (7d) by ${s}^{*}$. The solution Eqs. (7a, 7b, 7c, 7d) exists only for $s<{s}^{*}$.

In Figs. 3b, 4b, and 5b, we illustrate the case with positive $s$, which can be compared with Figs. 3a, 4a and 5a for $s=0$.

### Dependence on $\mathbf{s}$, Non-inflammatory Activation of Immunity

**Fig. 6:**
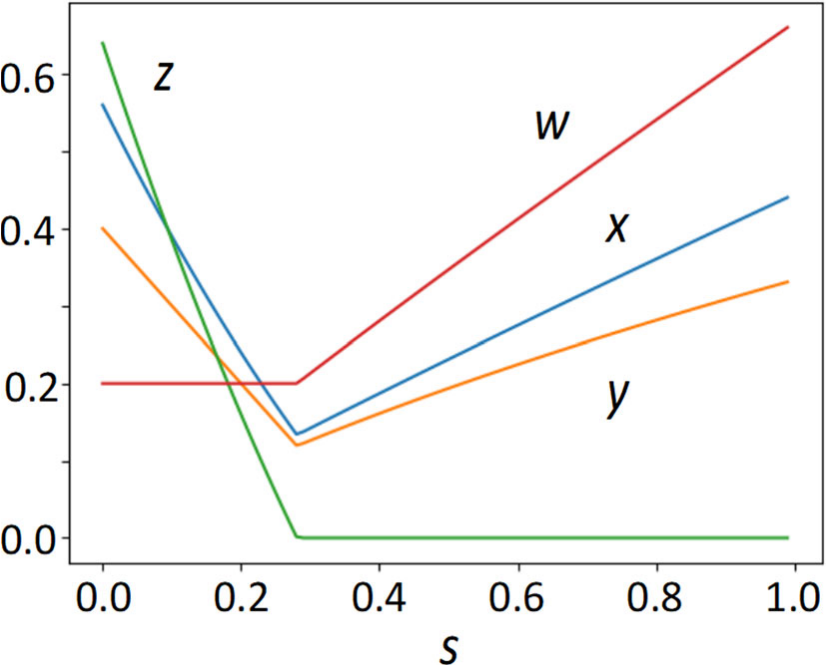
Dependence of variables at equilibrium on $s$, non-inflammatory immune activation. Tissue damage (blue); inflammation (orange); immune responses (red); pathogen abundance (green). The horizontal axis indicates $s$. As s increases, the pathogen abundance $z$ decreases and becomes zero for $s>{s}^{*}$, where ${s}^{*}\approx 0.275$. The tissue damage $x$ decreases with $s$ for $s<{s}^{*}$ but increases with $s$ for $s>{s}^{*}$, and it achieves the minimum when $s={s}^{*}$. Other parameters are: $a=0.2$, $b=0.2$, $c=0.2$, $d=0.2$, $e=0.2$, $f=1$, $g=0.3$, $h=2$, $k=2$, and $r=0.4$

Figure 6 illustrates the dependence of $x$, $y$ and $z$ on the magnitude of $s$, the non-inflammatory rate of immune activation (i.e., the basic defense provided by innate immunity). At $s={s}^{*}$, there is a transition from the presence of pathogen ($z>0$) to the absence of pathogen ($z=0$). We can see that the tissue damages $x$ should decrease with $s$ in $0<s<{s}^{*}$ but increases with $s$ in $s>{s}^{*}$. Hence, tissue damage is minimized when $s$ is the boundary between two parameter regions.

The condition for stability of the equilibrium with a positive abundance of pathogens is given in Appendix B (refer to Eq. (28).

Figure 3b illustrates the values of four variables at equilibrium when non-infectious activation exists ($s=0.1$). We may compare this result with Fig. 3a for the case of $s=0$, with the same values for the other parameters. In the case with $s>0$, the pathogen is absent ($z=0$) for a wider range of $d$; when $z>0$, the value of $z$ is smaller than the case with $s=0$. This is because the non-inflammatory activation of immunity would suppress the pathogen. On the other hand, tissue damage $x$ for small $d$ is larger for $s>0$ than for $s=0$. This is due to enhanced immune activity by non-inflammatory activation, leading to tissue damage.

Likewise, Fig. 4b shows bifurcation diagram of how pathogen abundance z changes with $d$ for $s>0$. We may compare this result with the one in Fig. 4a for $s=0$, which has the same values for the other parameters. The pathogen is absent for a wider range of $d$, and, when it is present, pathogen abundance is smaller for $s>0$. Oscillation of pathogen abundance for large $d$ requires even larger $d$ for $s>0$ than for $s=0$.

Figure 5b illustrates the phase plane when $s$ is positive ($s=0.5$). If we compare it with Fig. 5a for $s=0$, we reach a similar conclusion as in the last paragraph.

Overall, stronger non-inflammatory immune activation helps eliminate the pathogen from the body, reduces the pathogen abundance at equilibrium, and suppresses the tendency of oscillation with large amplitude. Under stronger $s$, tissue damage caused by persisting pathogens is reduced, but the damage caused by excess innate immunity is enhanced. Figure 6 shows that value of $s$ that minimizes the tissue damage $x$ is at the boundary between two parameter regions: one for $z=0$ and the other for $z>0$.

## Discussion

In this paper, we explored mathematical models depicting the coupled dynamics of innate immunity and inflammatory cytokine release following tissue damage. Our objective was to elucidate the diverse patterns of symptoms observed in diseases characterized by chronic inflammation.

The analysis of the model suggests that the inflammation triggered by the infection may persist even after pathogen is eliminated from the body. This "chronic inflammation" is more likely to persist with a stronger induction of inflammatory cytokines based on signals from damage (large $a$), stronger damage to tissues accompanying immune attacks (large $b$), and rapid induction of immunity by inflammatory cytokines (large $f$). In contrast, inflammation is less likely to persist at high level if recovery from damage occurs fast (large $g$), mortality of immune cells is fast (large $k$), and breakdown of inflammatory cytokines is rapid (large $d$). Considering these results, we hypothesize that, in conditions where chronic inflammation is prevalent, such as during aging, some parameters may shift in a way that favors the persistence of inflammation.

### Perpetual Oscillation

Furthermore, the model presented in this study demonstrates that the abundance of pathogens remaining in the model may not be in a stationary manner, but it could keep oscillating along with immune strength, tissue damages, and inflammation strength.

We analyzed parameter sensitivity of the period and amplitude of the oscillation in a case illustrated in Fig. 4b (please refer to Appendix C). The elasticity if each of 11 parameters in the model showed the following results: [1] the enhanced rates of decay and self-suppression of the variables (larger $d$, $e$, $g$) made the period of oscillation shorter. [2] the amplitude is highly sensitive to changes in the parameters. [3] The oscillation amplitude decreased with faster rates of interaction between variables (e.g., large $a$, $b$, $f$), which tended to stabilize the dynamics. In contrast, faster decay or decomposition of variables (e.g., large $d$, $g$, and $k$) destabilized the dynamics, resulting in larger amplitudes.

This could be interpreted as a contributing factor to the periodic symptoms observed in some inflammatory diseases. One of the factors contributing to ulcerative colitis, a disease characterized by chronic inflammation in the colon, is the recognition and attack of harmless microbes in the intestine as antigens (Ordás et al. 2012; Saez et al. 2023). In this context, these microbes can play the role depicted in our model as the pathogens. Additionally, ulcerative colitis exhibits a fluctuating pattern between active and remission phases, where inflammatory reactions oscillate, alongside cases where certain symptoms persist over the long term.

Innate immune responses may cause tissue damage that enhances inflammations, which in turn further enhances immune responses. The cycle of enhancement and activation of three reactions, along with nonlinear suppression of inflammation, leads to a finite level of enhancement, ultimately resulting in the eradication of pathogens from the body. This suggests that innate immunity, in conjunction with chronic inflammation, can suppress a variety of pathogens, ultimately leading to their eradication from the tissue.

Several chronic diseases exhibit oscillations, manifesting as periodic fluctuations in symptoms or disease progression (Banoth et al. 2016; Vanoni et al. 2016; Cudrici et al. 2020). For instance, rheumatoid arthritis, an autoimmune disease where the immune system attacks the joints, often manifests symptoms periodically. Patterns of occasional worsening of pain and joint swelling followed by improvement are observed (Baker et al. 2013). Epilepsy is a neurological disorder caused by abnormal electrical activity in the brain, resulting in seizures that occur periodically. The frequency and severity of seizures vary among individuals but may exhibit a certain degree of periodicity. Bronchial asthma is a condition characterized by difficulty breathing due to inflammation and constriction of the airways, with intermittent symptoms occurring occasionally. Symptoms may vary throughout the day, at night, or depending on the season. Chronic fatigue syndrome is a disease characterized by overall fatigue and decreased stamina, with symptoms that may fluctuate periodically. Patients may experience occasional relief from fatigue, while at other times, it may worsen. In these diseases, the fluctuation of periodic symptoms may impact the daily life and the management of treatment.

There have been many mathematical studies on oscillations generated by viral dynamics and the immune system (Nowak et al. 1995; Andreassen et al. 1997; Castillo-Chavez et al. 1989) or cancer coupled with the immune system (Eftimie et al. 2011; Kirschner and Panetta 1998; Mayer et al. 1995). All of these studies focus on the oscillatory behavior of immune responses, including adaptive immunity, whilst in this work we showed oscillation in dynamics of innate immunity and inflammation.

### Non-inflammatory Activation of Immune Reaction

We also analyzed the effect of non-inflammatory activation of immune reaction (denoted as $s$), which represents the baseline defense provided by innate immunity, capable of sustaining a positive level of protection even in the absence of pathogens. As non-inflammatory activation of the immune reaction becomes stronger, it influences the cyclic activation of the three processes: immunity, inflammation, and tissue damage.

A weak immune response allows for the large abundance of pathogens, which may result in a high tissue damage (Perelygina et al. 2020). On the other hand, an increase in immune strength beyond the level needed to eliminate pathogens leads to excess immune strength, which also cause tissue damage. Hence, the strength of immune responses achieves minimum tissue damage when it successfully exterminates the pathogen from the body. This observation is consistent with the choice of parameters, such as parameter $d$.

### Possibility of Eradication of Pathogens

The dynamics given by Eqs. (1a–1d) depict the interaction between innate immunity and inflammation, addressing phenomena before the onset of adaptive immunity. If the pathogens are not eradicated by innate immunity and remain in the body, adaptive immunity starts to function, which is outside the scope of the current paper. Pathogens can be eradicated by adaptive immunity, which is carried out by antigen-specific immune cells (e.g., helper T cells, cytotoxic T cells, B cells). However, pathogens can sometimes persist in the body even under the operation of adaptive immunity. Reasons for this persistence include the pathogen evading the immune response, forming reservoirs within the body, developing resistance to treatment, or the immune system being compromised and unable to effectively clear the infection. Examples of pathogens that can persist within the body include certain bacteria, such as *Mycobacterium tuberculosis*, which causes tuberculosis (Barry et al. 2009), and *Borrelia burgdorferi*, which causes Lyme disease (Steere 2001), as well as certain viruses, such as herpes simplex virus (Whitley and Roizman 2001), human immunodeficiency virus (Barre-Sinoussi and Montagnier 2003), hepatitis B virus (Rok and McMahon 2007), and hepatitis C virus (Davis and Baak 2004). In these cases, chronic or persistent infections can lead to long-term health complications, necessitating ongoing medical management to control the infection and prevent further spread or damage.

The model explains that tissue damage is caused by deficient or excessive immune responses, as shown in Figs. 3 and 6.

### Future Studies

Modeling adaptive immunity and the proliferation of pathogens, such as viruses in the host body, has been a central issue in the field of mathematical studies of immunology (Perelson and Nelson 1999; Nowak and May 2000). The dynamical models of immune cells in adaptive immunity and pathogens are somewhat similar to models in ecology. The emergence of pathogen mutants that evade immune responses resembles the processes described in population genetics theory (Nowak and May 2000; Grenfell et al. 2004).

However, recent advances in immunology suggest that the role of innate immunity is more significant than previously thought (Parham 2021). The innate immune response serves as the body's first line of defense against pathogen invasion or physical damage. Even more importantly, innate immunity is closely linked with chronic inflammation, and thus deserves greater attention in modeling efforts.

There have been mathematical and computational modeling of innate immunity and chronic inflammation, shedding light of aspects other than activation cycle studied in this study. Nakaoka et al. (2016) have addressed the competition between multiple bacterial strains. leading to chronic inflammation in epidemics, Abudukelimu et al. (2018) discussed on the switching between acute and chronic inflammation in computational models. Azher and Vodovotz (2014) reviewed mathematical modeling of innate immunity in diseases.

The model studied in this paper is a simple example illustrating the phenomena produced by the interaction between innate immunity and chronic inflammation. Even this straightforward work suggests a diversity of phenomena resulting from their interaction and highlights the potential for further studies. Our understanding of the molecular and cellular mechanisms underlying their interaction has developed rapidly. Given the critical role of innate immunity in protecting our body from pathogens and diseases, we believe that further studies on mathematical modeling of the interaction between innate immunity and chronic inflammation will deepen our understanding of the body’s defense mechanisms.

The behavior shown by the model in this paper exemplifies how dynamics involving positive and negative inflammation feedback loops can induce oscillations or nonlinear responses. The model assumes that suppression occurs when activation becomes excessive, represented as $e$. However, there may be responses not considered here that are involved. For instance, some inflammatory cytokine compartments can activate themselves (e.g., IL-6 amplifier; Hirano et al. 2021). In future studies, it will be important to consider and quantitatively evaluate such influences to determine what is crucial in defining the overall system's behavior. Addressing this will be a key topic for future research.

## Data Availability

Data sharing is not applicable to this article because no datasets were generated or analyzed during this study.

## Appendix A

In this Appendix, we examine the equilibria of the dynamics given by Eqs. (4), (1b), (1c) and (1d), where the immune responses are activated both by the basic level of activation $s$ and by inflammation at the rate proportional to its strength, $fy$. For the case where activation is proportional to inflammation, given by Eq. (1a) instead of Eq. (4), we can obtain the results by setting $s$ equal to zero.

We first consider the equilibria of the four-variable dynamics, given by Eqs. (4), (1b). (1c), and (1d). The following equations hold:

8a $$0=s+fy-kw$$

8b $$0=bw+cz-gx$$

8c $$0=ax-\left(d+ey\right)y$$

8d $$0=\left[r-hw\right]z$$

Equations (8d) indicates that either $z=0$ or $r-hw$ holds. In the latter case, $z>0$ needs to be satisfied. In the following, we examine these two cases in this order.

### Equilibrium Without Pathogen (${\varvec{z}}=0$)

From Eq. (8a) we have

9 $$w=\frac{1}{k}\left(s+fy\right)$$

In the equilibrium without pathogens ($z=0$), the following equations hold:

10a $$0=\frac{b}{k}\left(s+fy\right)-gx$$

10b $$0=ax-\left(d+ey\right)y$$

By eliminating $x$ from Eqs. (10a) and (10b), we have

11 $$\frac{b}{gk}\left(s+fy\right)=\frac{1}{a}\left(d+ey\right)y$$

which is rewritten as follows:

12 $$e{y}^{2}+\left(d-\frac{abf}{gk}\right)y-\frac{abs}{gk}=0$$

When $s>0$, Eq. (12) has the following positive solution:

13 $$y=\frac{1}{2e}\left(\frac{abf}{gk}-d+\sqrt{{\left(\frac{abf}{gk}-d\right)}^{2}+4\frac{eabs}{gk}}\right)$$

From this, we can calculate two other variables as follows:

14 $$ w = \frac{1}{k}\left( {s + fy} \right)\;{\text{and}}\;x = \frac{b}{gk}\left( {s + fy} \right) $$

both of which increase with $y$.

Concerning the dependence of these variables on $s$, All the three variables, $y, w, x$, increase with $s$.

#### When the Non-inflammatory Activation Rate is Absent (${\varvec{s}}=0$)

When $s=0$, Eq. (12) has two solutions:

15 $$ y = 0\;{\text{and}}\;y = \frac{1}{e}\left( {\frac{abf}{{gk}} - d} \right) $$

When $y=0$ holds, all the other variables are zero: ($z=0, x=0, w=0$). However, this equilibrium is always unstable, as confirmed by the linearized dynamics of $z$ around the origin, given by $\frac{dz}{dt}\approx rz$ due to $w=0$.

Hence, in this section, we focus on the equilibrium with $y=\frac{1}{e}\left(\frac{abf}{gk}-d\right)$, which is equal to the limit of the formula Eq. (11) when $s$ converges to 0. From Eq. (14) with $s=0$, we have $w=\frac{f}{k}y$ and $x=\frac{bf}{gk}y$, which leads to the following equations:

16 $$ w = \frac{f}{ke}\left( {\frac{abf}{{gk}} - d} \right),\;{\text{and}}\;x = \frac{bf}{{gke}}\left( {\frac{abf}{{gk}} - d} \right) $$

These are derived from Eqs. (8a) and (8c), together with $z=0$.

The equilibrium obtained in this way may or may not be stable. Stability of the equilibria are discussed below.

### Equilibrium with Some Pathogens (${\varvec{z}}>0$)

Next, we consider the cases with some pathogens are maintained. From Eq. (8d) and $z>0$, $w=\frac{r}{h}$ holds. From Eq. (8a), we have $y=\frac{1}{f}\left(\frac{rk}{h}-s\right)$. Then Eq. (8c) is rewritten as follows:

17a $$x=\frac{1}{a}\left(d+\frac{e}{f}\left(\frac{rk}{h}-s\right)\right)\frac{1}{f}\left(\frac{rk}{h}-s\right)$$

From Eq. (8b), we have

17b $$z=\frac{1}{c}\left(gx-bw\right)=\frac{1}{c}\left(\frac{g}{a}\left(d+\frac{e}{f}\left(\frac{rk}{h}-s\right)\right)\frac{1}{f}\left(\frac{rk}{h}-s\right)-\frac{br}{h}\right)$$

Hence, we have the following expression of the equilibrium with $z>0$:

$$w=\frac{r}{h}$$, $$y=\frac{1}{f}\left(\frac{rk}{h}-s\right)$$, $$x=\frac{1}{a}\left(d+\frac{e}{f}\left(\frac{rk}{h}-s\right)\right)\frac{1}{f}\left(\frac{rk}{h}-s\right)$$,

18 $$ {\text{and}}\;z = \frac{1}{c}\left( {\frac{g}{a}\left( {d + \frac{e}{f}\left( {\frac{rk}{h} - s} \right)} \right)\frac{1}{f}\left( {\frac{rk}{h} - s} \right) - \frac{br}{h}} \right) $$

#### Equilibrium with Pathogen when Non-inflammatory Activation is Absent (${\varvec{s}}=0$)

When the activation of immune responses is simply proportional to the inflammation ($s=0$), the equilibrium with some pathogen becomes as follows:

19 $$ w = \frac{r}{h},\;y = \frac{rk}{{hf}},\;x = \frac{1}{a}\left( {d + e\frac{rk}{{hf}}} \right)\frac{rk}{{hf}},\;{\text{and}}\;z = \frac{1}{c}\left( {\frac{g}{a}\left( {d + e\frac{rk}{{hf}}} \right)\frac{rk}{{hf}} - b\frac{r}{h}} \right) $$

For the abundance of pathogen at the equilibrium $z$ to be positive, the following inequality must hold:

20 $$d+e\frac{rk}{hf}>\frac{abf}{gh}$$

If the inequality opposite to Eq. (13) holds, no equilibrium with $z>0$ exists.

## Appendix B

In this Appendix, we examine the local stability of the equilibria, obtained in Appendix A.

### Stability of the Equilibria in the Reduced Three Variable Dynamics

We first discuss the stability in the reduced dynamics with three variables in which the immune response $w$ converges quickly to the quasi-equilibrium much faster than the other three variables ($x, y,$ and $z$). Using $w=\frac{1}{k}\left(s+fy\right)$, the dynamics of the three other variables become

21a $$\frac{dx}{dt}=\frac{b}{k}\left(s+fy\right)+cz-gx$$

21b $$\frac{dy}{dt}=ax-\left(d+ey\right)y$$

21c $$\frac{dz}{dt}=\left[r-\frac{h}{k}\left(s+fy\right)\right]z$$

We first analyze the stability of the equilibria of the dynamics. Then we compared the results with behavior of the original dynamics with four variables, given as Eq. (1).

Here, we study the stability of the equilibrium where some pathogens remain. For simplicity of the analysis, we consider the reduced dynamics with three variables. The dynamics of three variables ($x, y,$ and $z$) are given by Eqs. (21a, 21b, 21c). The characteristic equation for the eigenvalues around the equilibrium $(x, y, z)$ is given as follows:

22 $$0=det\left\Vert \begin{array}{ccc}-g-\lambda & \frac{bf}{k}& c\\ a& -\left(d+2e\overline{y }\right)-\lambda & 0\\ 0& -\frac{hf}{k}\overline{z }& r-\frac{h}{k}\left(s+f\overline{y }\right)-\lambda \end{array}\right\Vert $$

#### Stability of the Equilibrium Without Pathogen $\overline{z }=0$

We first consider the equilibrium without pathogen ($\overline{z }=0$). The values of the equilibrium are given by Eqs. (13) and (14). Characteristic equation Eq. (22) becomes

23 $$0=-\left(\lambda -r+\frac{hs}{k}+\frac{hf}{k}\overline{y }\right)\left[\left(\lambda +g\right)\left(\lambda +d+2e\overline{y }\right)-\frac{abf}{k}\right]$$

The local stability condition is

24a $$r<\frac{hs}{k}+\frac{hf}{k}\overline{y }$$

24b $$ {\text{and}}\;g\left( {d + 2e\overline{y}} \right) > \frac{abf}{k} $$

The latter inequality Eq. (24b) is from the Routh-Hurwitz condition for the second factor of Eq. (23) to have both roots with negative real parts. The second inequality of Eq. (24b) holds for the equilibrium with positive pathogen abundance (see the last section). If the first inequality holds, the equilibrium is stable, and pathogens invading the system with a small quantity cannot increase in the system. From Eq. (12) that holds on this equilibrium, we have $ge{\overline{y} }^{2}+gd\overline{y }-\frac{abf}{k}\overline{y }-\frac{abs}{k}=0$, which leads to $g\left(d+2e\overline{y }\right)=ge\overline{y }+\frac{abf}{k}+\frac{abs}{k\overline{y} }>\frac{abf}{k}$, implying that inequality given by Eq. (24b) always holds.

Hence, the stability of the equilibrium without pathogen is as follows:

25a $$ {\text{It}}\;{\text{ is }}\;{\text{stable}},\;{\text{ if}}\;r < \frac{hs}{k} + \frac{hf}{k}\overline{y}, $$

25b $$ {\text{It }}\;{\text{is }}\;{\text{unstable}},\;{\text{ if}}\;r > \frac{hs}{k} + \frac{hf}{k}\overline{y}, $$

In the latter situation, the equilibrium $\overline{z }=0$ is unstable, and it can be invaded by the pathogen of a small amount.

#### Stability of the Equilibrium with Pathogen $\overline{z }>0$

The stability of the equilibrium with positive abundance of pathogen is also calculated from Eq. (22). However, the term including $\overline{z }$ does not vanish in this case.

By noting that $r-\frac{h}{k}\left(s+f\overline{y }\right)=0$ holds at this equilibrium, the characteristic equation given by Eq. (22) becomes

$$0=det\left\Vert \begin{array}{ccc}-g-\lambda & \frac{bf}{k}& c\\ a& -\left(d+2e\overline{y }\right)-\lambda & 0\\ 0& -\frac{hf}{k}\overline{z }& -\lambda \end{array}\right\Vert $$

26 $$=-\lambda \left(\lambda +g\right)\left(\lambda +d+2e\overline{y }\right)+ac\frac{hf}{k}\overline{z }-\lambda a\frac{bf}{k}$$

which leads to the following equation:

$$\lambda \left(\lambda +g\right)\left(\lambda +d+2e\overline{y }\right)-a\frac{bf}{k}\lambda +\frac{hf}{k}\overline{z }ac=0$$

which is rewritten as

27 $${\lambda }^{3}+\left(g+d+2e\overline{y }\right){\lambda }^{2}+\left(\left(d+2e\overline{y }\right)g-\frac{abf}{k}\right)\lambda +\frac{hf}{k}\overline{z }ac=0$$

According to Rough-Hurwitz condition, the local stability of the equilibrium (i.e. all the eigenvalues have negative real parts) is

28 $$\left(g+d+2e\overline{y }\right)\left(\left(d+2e\overline{y }\right)g-\frac{abf}{k}\right)>\frac{hf}{k}\overline{z }ac$$

For simplicity, if we consider the case in which the immune activation is proportional to the inflammation ($s=0$), the location of the equilibrium is given by Eq. (19). The stability condition Eq. (28) becomes as follows:

29 $$\left(g+d+2\frac{erk}{hf}\right)\left(\left(d+2\frac{erk}{hf}\right)g-\frac{abf}{k}\right)>\frac{hf}{k}ac\frac{1}{c}\left(\frac{g}{a}\left(d+\frac{erk}{hf}\right)\frac{rk}{hf}-\frac{br}{h}\right)=\left(gr\left(d+\frac{erk}{hf}\right)-\frac{abfr}{k}\right)$$

which is Eq. (4) in text.

For $s>0$, the condition is Eq. (28) combined with Eq. (18), which becomes messier than Eq. (29) for $s=0$. Calculation from Eq. (28) with the equilibrium numerically replaced would be more practical than explicit formula.

By numerical analysis of the dynamics directly, we confirmed Eqs. (28) and (29) for the stability condition of the positive equilibrium.

### Stability of the Equilibrium in Four-Variable Dynamics

We consider the stability of the original dynamics with four variables:

30a $$\frac{dw}{dt}=s+fy-kw$$

30b $$\frac{dx}{dt}=bw+cz-gx$$

30c $$\frac{dy}{dt}=ax-\left(d+ey\right)y$$

30d $$\frac{dz}{dt}=\left[r-hw\right]z$$

#### Stability of the Equilibrium Without Pathogen

We first consider the stability of the equilibrium without pathogen ($\widehat{z}=0$).

$$\frac{\partial }{\partial w}\frac{dw}{dt}=-k$$, $$\frac{\partial }{\partial x}\frac{dw}{dt}=0$$, $$\frac{\partial }{\partial y}\frac{dw}{dt}=f$$, $$\frac{\partial }{\partial z}\frac{dw}{dt}=0$$,

$$\frac{\partial }{\partial w}\frac{dx}{dt}=b$$, $$\frac{\partial }{\partial x}\frac{dx}{dt}=-g$$, $$\frac{\partial }{\partial y}\frac{dx}{dt}=0$$, $$\frac{\partial }{\partial z}\frac{dx}{dt}=c$$,

$$\frac{\partial }{\partial w}\frac{dy}{dt}=0$$, $$\frac{\partial }{\partial x}\frac{dy}{dt}=a$$, $$\frac{\partial }{\partial y}\frac{dy}{dt}=-d-e\widehat{y}$$, $$\frac{\partial }{\partial z}\frac{dy}{dt}=0$$,

$\frac{\partial }{\partial w}\frac{dz}{dt}=0$, $\frac{\partial }{\partial x}\frac{dz}{dt}=0$, $\frac{\partial }{\partial y}\frac{dz}{dt}=0$, $\frac{\partial }{\partial z}\frac{dz}{dt}=r-h\widehat{w}$,where we let $Q=d+e\widehat{y}>0$. The characteristic equation is:

$$0=det\left\Vert \begin{array}{cccc}-k-\lambda &0 &f&0\\ b& -g-\lambda & 0 &c\\ 0 & a & -Q-\lambda &0 \\ 0& 0 & 0 & r-h\widehat{w}-\lambda \end{array}\right\Vert $$

31 $$=\left(r-h\widehat{w}-\lambda \right)det\left\Vert \begin{array}{ccc}-k-\lambda & 0& f\\ b& -g-\lambda & 0\\ 0& a& -Q-\lambda \end{array}\right\Vert $$

The four roots are:

$\lambda =r-h\widehat{w}$ and three roos of $\left(-k-\lambda \right)\left(-g-\lambda \right)\left(-Q-\lambda \right)+abf=0$

The latter becomes ${\lambda }^{3}+\left(k+g+Q\right){\lambda }^{2}+\left(kg+gQ+kQ\right)\lambda +kgQ-abf=0$

From Eq. (11), we have $Q=d+ey=\frac{b}{gk}\left(s+f\widehat{y}\right)\frac{a}{y}=\frac{abs}{gk\widehat{y}}+\frac{abf}{gk}$. Hence,

$$kgQ-abf=\frac{abs}{\widehat{y}}>0$$.

The Rough-Hurwitz condition for the second polynomial is:

$$\left(k+g+Q\right)\left(kg+gQ+kQ\right)$$> $$kgQ-abf$$

We can show that this inequality always hold.

Hence, the stability for the equilibrium is $r<h\widehat{w}$. If this holds, rare pathogen invading the body with a small initual abundance would increase. If instead $r>h\widehat{w}$, the pathogen with a low abundance would increases and infect the host.

#### Stability of the Equilibrium Without Pathogen

At the equilibrium with some pathogen ($\widehat{z}>0,\text{and} r=h\widehat{w})$, we have

$\frac{\partial }{\partial w}\frac{dz}{dt}=-h\widehat{z}$, add $\frac{\partial }{\partial z}\frac{dz}{dt}=0$, and the characteristic equation becomes

$$0=det\left\Vert \begin{array}{cc}\begin{array}{cc}-k-\lambda & 0\\ b& -g-\lambda \end{array}& \begin{array}{cc} f& 0\\ 0& c\end{array}\\ \begin{array}{cc}0 & a\\ -h\widehat{z} & 0\end{array}& \begin{array}{cc}-Q-\lambda & 0 \\ 0& -\lambda \end{array}\end{array}\right\Vert $$

$$=\left(-k-\lambda \right)\left(-g-\lambda \right)\left(-Q-\lambda \right)\left(-\lambda \right)+fdet\left\Vert \begin{array}{ccc}b& -g-\lambda & c\\ 0& a& 0\\ -h\widehat{z}& 0& -\lambda \end{array}\right\Vert $$

$$=\left(\lambda +k\right)\left(\lambda +g\right)\left(\lambda +Q\right)\lambda +f\left(-ab\lambda +h\widehat{z}ac\right)$$

32 $$={\lambda }^{4}+\left(k+g+Q\right){\lambda }^{3}+\left(kg+kQ+gQ\right){\lambda }^{2}+\left(kgQ-abf\right)\lambda +fh\widehat{z}ac$$

All of this polynomial equation have negative real parts ($Re\left[\lambda \right]<0$) when the following Routh-Hurwitz condition holds:

33a $$ a_{1} > 0,\;a_{2} > 0,\;a_{3} > 0\;{\text{and}}\;a_{4} > 0 $$

33b $$ a_{1} a_{2} - a_{3} > 0,\;{\text{and}} $$

33c $$ a_{3} \left( {a_{1} a_{2} - a_{3} } \right) > a_{1}^{2} a_{4} $$

where we set

33d $$ a_{1} = k + g + Q,\;a_{2} = kg + kQ + gQ,\;a_{3} = kgQ - abf,\;{\text{and}}\;a_{4} = fh\hat{z}ac $$

(Gantmacher 1959). From Eqs. (33d), (33a) holds always except for ${a}_{3}>0$.

From ${a}_{4}>0$, the right-hand side of Eq. (33c) is positive. Given Eq. (33b), ${a}_{3}>0$ holds if Eq. (33c) holds. Hence, we have inequalities (33b) and (33c) for the stability of the equilibrium.

34 $${a}_{1}{a}_{2}-{a}_{3}=\left(k+g+Q\right)\left(kg+kQ+gQ\right)-kgQ+abf$$

Note that the first term on the right-hand side includes $kgQ$, Hence Eq. (33b) holds. Hence, the stability condition is a single inequality given as follows:

35 $$\left(kgQ-abf\right)\left(\left(k+g+Q\right)\left(kg+kQ+gQ\right)-kgQ+abf\right)>{\left(k+g+Q\right)}^{2}fh\widehat{z}ac$$

If the inequality opposite to Eq. (35) holds, the equilibrium is unstable. The inequality condition gives the stability condition if we replace the equilibrium $\widehat{z}$ either by mathematical formula, given by Eq. (18), or simply by numerical value.

## Appendix C

Here, we explain the procedure for evaluating the elasticity of the oscillation period. We selected a parameter set that generates perpetual oscillations. Using this set, we numerically determined the oscillation period, which depends on 11 parameters: ${q}_{1}$, ${q}_{2}$, ${q}_{3}$, …, ${q}_{11}$, representing $a$, $b$, $c$, $d$, $e$, $f$, $g$, $h$, $k$, $r$, and $s$, respectively. Each parameter ${q}_{i}$ was modified slightly, and the resulting change in the oscillation period was calculated to assess the magnitude of its effect. The sensitivity of the oscillation period to each parameter is quantified using elasticity, denoted as ${k}_{i}$, which represents the elasticity of the period with respect to parameter ${q}_{i}.$ It is defined as ${k}_{i}= \partial log \left[\text{period}\right]/\partial log{q}_{i} = {q}_{i}/\left[\text{period}\right]\bullet \partial \left[\text{period}\right]/\partial {q}_{i}$, for $i =$ 1, 2,…, 11. This index is independent of the choice of “unit” of each quantity and has been widely used to represent parameter sensitivity in fields such as economics and ecology (de Kroon et al. 1986; Acemoglu 2002; Hayashi et al. 2024).

We calculated the elasticity of the oscillation period for the case illustrated in Fig. 4b at $$d=0.35$$, which is near the bifurcation point ($$d=0.32)$$, with all the other parameters were kept the same as in Fig. 4b. The results were: $${k}_{a}=-0.10$$, $${k}_{b}=-0.08$$, $${k}_{c}=0$$, $${k}_{d}=-0.33$$, $${k}_{e}=-0.56$$, $${k}_{f}=0.46$$, $${k}_{g}=-0.64$$, $${k}_{h}=0.38$$, $${k}_{k}=-0.66$$, $${k}_{r}=0.64$$, and $${k}_{s}=-0.18$$. The period of oscillation decreased for larger $$d$$, $$e$$, $$g$$, and $$k$$ and increased for larger $$f$$ and $$r$$. This indicates that the enhanced rates of decay and self-suppression of the variables (larger $$d$$, $$e$$, $$g$$, and $$k$$) made the period of oscillation shorter.

We also calculated the elasticity of the amplitude of oscillation, ${k}_{i}= \partial log \left[\text{amplitude}\right]/\partial log{q}_{i}$, for $i =$ 1, 2,…, 11. The results were: ${k}_{a}=-5.56$, ${k}_{b}=-4.56$, ${k}_{c}=-1.00$, ${k}_{d}=2.62$, ${k}_{e}=-2.25$, ${k}_{f}=-3.32$, ${k}_{g}=3.19$, ${k}_{h}=-0.77$, ${k}_{k}=3.18$, ${k}_{r}=8.97$, and ${k}_{s}=-1.95$. Magnitudes of these values are quite large, indicating that the amplitude is highly sensitive to changes in the parameters. The oscillation amplitude decreased with faster rates of interaction between variables (e.g., large $a$, $b$, $f$), which tended to stabilize the dynamics. In contrast, faster decay or decomposition of variables (e.g., large $d$, $g$, and $k$) destabilized the dynamics, resulting in larger amplitudes.

A more detailed analysis of parameter sensitivity would be valuable for gaining a deeper understanding of the nature of the oscillations.
